# Psychometric evaluation of a culturally adapted illness perception questionnaire for African Americans with type 2 diabetes

**DOI:** 10.1186/s12889-022-13172-2

**Published:** 2022-04-13

**Authors:** Olayinka O. Shiyanbola, Deepika Rao, Sierra Kuehl, Daniel Bolt, Earlise Ward, Carolyn Brown

**Affiliations:** 1 Division of Social and Administrative Sciences, School of Pharmacy, University of Wisconsin-Madison, 777 Highland Avenue, Madison, WI 53705 USA; 2 Department of Educational Psychology, University of Wisconsin-Madison, Madison, WI USA; 3 Department of Nursing, University of Wisconsin-Madison, Madison, WI USA; 4 Division of Health Outcomes and Pharmacy Practice, University of Texas- Austin, Austin, TX USA

**Keywords:** Illness Perception Questionnaire, African Americans, Diabetes, Validity, Reliability, Psychometric analysis

## Abstract

**Background:**

Diabetes is burdensome to African Americans, who are twice as likely to be diagnosed, more likely to develop complications and are at a greater risk for death and disability than non-Hispanic whites. Medication adherence interventions are sometimes ineffective for African Americans because their unique illness perceptions are not adequately addressed. The Illness Perception Questionnaire-Revised (IPQ-R) that assesses illness perceptions has shown reliability and validity problems when used with African Americans. Thus, the study objective was to adapt the IPQ-R for African Americans and assess the validity and reliability of the culturally adapted questionnaire.

**Methods:**

The parent study used an exploratory sequential mixed methods design, to explore African Americans’ illness perceptions qualitatively, used the results to adapt the IPQ-R, and tested the culturally adapted IPQ-R items quantitatively. In this paper, a preliminary culturally adapted IPQ-R refined based on the qualitative study, was administered to 170 middle-aged United States-based African Americans with type 2 diabetes in a face-to-face survey. Content, construct, convergent, and predictive validity, including reliability was examined. Pearson and item-total correlations, item analysis, exploratory factor analysis, multiple linear regression analysis, and test-retest were conducted.

**Results:**

A revised culturally adapted IPQ-R was identified with a 9-factor structure and was distinct from the old factor structure of the original IPQ-R. The ‘consequences’ domain from the IPQ-R occurred as two factors (external and internal consequences) while the ‘emotional representations’ domain in the IPQ-R emerged as separate ‘present’ and ‘future’ emotional representation factors. Illness coherence’ was differently conceptualized as ‘illness interpretations’ to capture additional culturally adapted items within this domain. Most items had factor loadings greater than 0.4, with moderate factor score correlations. Necessity and concern beliefs in medicines significantly correlated with domains of the culturally adapted IPQ-R. Pearson’s correlation values were not greater than 0.7, indicating good convergent validity. The culturally adapted IPQ-R significantly predicted medication adherence. None of the correlation values were higher than 0.7 for the test-retest, indicating moderate reliability. Most domains of the culturally adapted IPQ-R had Cronbach’s alpha values higher than 0.7, indicating good internal consistency.

**Conclusions:**

The results provide preliminary support for the validity of the culturally adapted IPQ-R in African Americans with diabetes, showing good construct, convergent and predictive validity, as well as reliability.

**Supplementary Information:**

The online version contains supplementary material available at 10.1186/s12889-022-13172-2.

## Background

Type 2 diabetes has become highly burdensome in the United States for those diagnosed with the disease [1]. African Americans have almost a 2-fold higher prevalence of type 2 diabetes compared to non-Hispanic whites and bear a disproportionate burden of morbidity and mortality [2]. Moreover, African Americans with type 2 diabetes have two to four times higher rates of developing diabetes complications such as kidney failure, blindness, and lower limb amputations [3]. These increased diabetes complications may be due to lower diabetes medication adherence rates among African Americans compared to non-Hispanic whites. However, current interventions for improving lower medication adherence rates are not effective for African Americans, possibly because their unique illness perceptions are not adequately addressed or studied [4, 5]. Prior research shows that illness perceptions are directly related to medication adherence [6], so it is important to assess illness perceptions to improve medication adherence for African Americans.

In our prior qualitative research based on the illness perception self-regulatory model, we explored the perceptions of African Americans related to diabetes and found that their illness perceptions are intertwined with sociocultural and psychosocial factors, which may not be reflected in the current Illness Perception Questionnaire [7, 8]. One study in older African American women with depression found that religious beliefs and practices were the primary modes of coping with depression, as well as other culture-specific coping behaviors [9]. In our prior study, African Americans perceived that they developed diabetes due to a race-mediated effect related to past slavery and poverty, which influenced unhealthy eating habits and led to a diabetes diagnosis [7]. As well, there was a perception that diabetes made the family bonding experience during mealtimes difficult, and the disease diminished their cultural experiences and faith in God. However, positive thinking about survival influenced their perception of control. This previous research showed how African Americans’ sociocultural beliefs may influence their overall perceptions of diabetes and highlighted the need for further study of illness perceptions among African Americans.

The self-regulatory model designed by Leventhal et al., describes the process by which people respond to a health threat and have perceptions of their illness [10]. The model describes five dimensions within the cognitive representation of illness including identity, consequences, cause, timeline, cure, and control [10]. Research has showed the importance of illness perceptions to patient behavior, whereby, changing patients’ illness perceptions can improve diabetes self-management practices such as adherence [11]. The Illness Perception Questionnaire (IPQ) assesses the five cognitive illness representations using a five-point Likert scale [12]. The IPQ was later revised by extending the original scale and adding other items and domains, leading to about 80 items in total [13]. Subsequently, the IPQ was revised to form the Illness Perception Questionnaire-Revised (IPQ-R), which is more commonly used.

The IPQ-R is a reliable and valid scale that assesses illness perceptions and is used to explain patient behaviors in different chronic illnesses including diabetes [14]. Illness perceptions are particularly associated with medication use behaviors, such as medication adherence [6]. Although the IPQ-R is a widely used standardized measure for assessing illness perceptions, it was validated in Western European populations, causing significant reliability and validity measurement problems when used with individuals of African descent [15]. Abubakari et al. investigated the internal reliability of the IPQ-R and suggested further evaluation before using the IPQ-R in other cultural groups. They recommended addressing cultural issues to enhance understanding and interpretation of the questions among racial/ethnic groups [15]. The IPQ-R does not account for familial relationships, racial identity, and religious beliefs, despite the importance of these factors to African Americans’ illness perceptions [7] . In one study, five of the IPQ-R subscales had consistency issues among African Americans with mental illness in relation to their coping behavior [16]. The psychometric issues observed in assessing beliefs about mental illness among African Americans may also occur when assessing beliefs about diabetes among African Americans. Another study noted that when using the IPQ-R in African Americans with mental illness, the internal reliability was much lower [17]. Finally, Calvin et al. found that the risk perception for diabetes complications in African Americans was not related to many of the IPQ-R domains (except identity, emotion, and consequences) [18].

### Objective

Given the identification of measurement issues with the use of the IPQ-R in assessing illness perceptions in African Americans, the purpose of this study was to adapt the IPQ-R for African Americans with diabetes to account for their unique illness perceptions. Consequently, this study assessed the validity and reliability of the culturally adapted questionnaire.

### Methods

#### Study Design

This research was conducted in three phases using an exploratory sequential mixed methods design. We explored African Americans’ illness perceptions in the qualitative phase (Phase 1) [7, 8], added 44 new culturally-adapted items to the IPQ-R based on the qualitative findings, and validated the content of the IPQ-R via cognitive interviewing with African Americans with diabetes (Phase 2) [19]. Finally, we compared the qualitative and quantitative findings [20], and tested the validity and reliability of the culturally adapted IPQ-R in the quantitative phase (Phase 3). This paper reports the results of Phase 3: the validity and reliability testing of the questionnaire. The mixed methods design was important for adapting and testing a questionnaire that would identify important sociocultural aspects influencing the illness perceptions of African Americans with diabetes. Evidence collected in support of the validity and reliability of a questionnaire should first and foremost address its intended use and the sources of measurement error most likely to impact its use. Therefore, the psychometric analysis was designed to evaluate content validity, criterion-related validity, construct validity, and quantify the measurement error in both internal consistency and test-retest reliability coefficients. We also evaluated how the illness perceptions in the questionnaire are associated with African Americans’ medication adherence (an outcome measure), i.e., the predictive validity of the culturally adapted IPQ-R.

#### Sample and Recruitment

A convenience and snowball sample of middle-aged United States-based African American men and women in a Midwestern state, 45–60 years old, with type 2 diabetes at least one-year prior, and who took at least one oral prescription diabetes medication was used for the surveys. Participants self-reported if they were diagnosed with diabetes. We did not determine participant’s ethnicity such as whether they were Puerto Rican, Mexican, Haitian, Jamaican, or Black Cubans (Hispanic origin).

The exclusion criteria were not self-identifying as African American/Black, younger than 45 years and older than 60 years and using only injectable insulin. The inclusion/exclusion criteria were kept identical to the criteria for the prior qualitative phase to ensure good integration between the two phases. Survey participants were recruited using convenience sampling from food pantries, churches, diabetes support groups, apartment complexes, and clinics in a Mid-Western State in the United States. Flyers, personal contact, and word of mouth were the main recruitment strategy. As well, we used snowballing sampling where we asked other individuals who participated in the survey to recommend other individuals they know who fit the same inclusion criteria and could participate in the study. Surveys were completed in-person in a face-to-face survey.

A subset of the original sample completed the follow-up survey. Participants received $25 for completion of the baseline survey and $30 for the follow-up survey. The study was approved by the Institutional Review Board at the principal investigators’ university. Informed consent was obtained from all participants. All methods were carried out in accordance with relevant guidelines and regulations.

### Data Collection

#### Qualitative

In prior studies, we initially conducted six focus groups with a sample of African Americans with type 2 diabetes who fit the same inclusion and exclusion criteria. Guided by a phenomenology approach, a discussion was facilitated by a PhD trained individual with extensive experience in qualitative research. Question domains explored perceptions of diabetes based on Levanthal’s self-regulatory model. Subsequently, we used the qualitative findings to create new culturally adapted items, added to the IPQ-R and refined the content of the new adapted items using cognitive interviewing with African Americans with diabetes. This work is published elsewhere [7, 8, 19].

#### Questionnaire

The quantitative phase involved psychometric testing of the newly developed, culturally adapted IPQ-R through a survey that included demographic information, original IPQ-R items, the Beliefs in Medicines scale [21], and the Adherence to Refills and Medication-Diabetes (ARMS-D) scale [22]. The IPQ-R has three components including the individual’s beliefs about the illness, the identity or labels through which the patient connects themselves with the illness and causes (attribution of the illness). Other than the identity and cause component, there are seven subscales within the individual beliefs about illness component. Due to the long length of the original IPQ-R survey and to minimize responder burden, we only included items from the original questionnaire that was not adapted in our culturally adapted IPQ-R. The Beliefs in Medicines scale has two sub-scales: concern and necessity beliefs about medicines. The ARMS-D scale measures self-reported medication adherence among patients with diabetes.

The face-to-face survey took 15–20 min to complete. The survey was administered at baseline and 6–8 weeks later at follow-up to a subset of respondents who agreed to complete the survey again later to assess test-retest reliability. The items in the follow-up survey were identical to the baseline survey, except for the demographic and clinical information which were collected only in the baseline survey.

### Data Analysis

#### Face and Content Validity

The representativeness of questionnaire items in relation to African Americans’ beliefs about diabetes was assessed using face and content validation procedures. Content validation was conducted based on the input of two experts familiar with the illness perceptions construct. These subject-matter experts provided feedback on how well each question measured the intended construct. The experts, who completed face validity, included individuals who were African American and had expertise in psychology and patient illness and treatment decisions. Each questionnaire item was examined according to its accuracy and importance in the measurement of African Americans’ beliefs. To avoid construct overrepresentation, we evaluated and deleted any items that should not be included in the questionnaire. To avoid construct underrepresentation, we checked for a comprehensive representativeness of the content in the questionnaire.

#### Construct Validity

To evaluate the ability of the culturally adapted IPQ-R to measure its intended construct, we used exploratory factor analysis procedures [23]. Item factor analysis provided a basis for evaluating whether the inter-correlations among questionnaire items and the factor structure resembles those consistent with the underlying core illness perceptions (including items in the new sociocultural influences domain). Items with poor inter-correlations were re-evaluated and, in many instances, removed from further analysis. Questionnaire items were also assessed for effectiveness by observation of a factor loading greater than 0.4. Items with factor loadings less than 0.4 were retained but will be re-assessed in future studies. Factor score inter-correlations were used to evaluate distinguishability of the factors as well as lend insight into potential overlap between illness perceptions. We used a maximum likelihood method with Promax rotation and Kaiser Normalization to achieve the factor structure that best matched theoretical conceptualizations. Maximum likelihood tends to be the default approach in exploratory factor analysis due to its attractive statistical properties, and tends to outperform competitors (e.g., principal axis factoring) when there is substantial variability in loadings across indicator variables, as seems to be the case in our analyses [24]. Our use of Promax rotation is consistent with our expectation that the most interpretable factors underlying our instrument will likely correlate.

#### Convergent Validity

At the scale score level, we established the convergent validity of the culturally-adapted IPQ-R by assessing the relationship between illness perceptions from the adapted IPQ-R and beliefs in medicine from the Beliefs in Medicine Questionnaire [21]. Convergent validity is a sub-type of construct validity that examines two measures that are supposed to be measuring the same construct and shows that they are related. Based on the self-regulatory model, illness perceptions would moderately correlate with beliefs in medicines if there is convergence [25, 26]. We used Pearson correlations to evaluate these relationships and examine convergent validity [27].

#### Predictive Validity

A multiple linear regression was used to examine if illness perceptions from the culturally adapted questionnaire predicted medication adherence [28, 29]. The regression analysis reflects the validation evidence of the most critical form for the adapted questionnaire, as the ultimate objective in measuring illness perceptions is to influence medication adherence. Medication adherence was chosen as the outcome based the literature that suggests that illness perceptions are associated with medication adherence, an outcome measure that assesses medication use in diabetes [30, 31] and other chronic illnesses [32–36]. The analysis was used to determine if the illness perception subscales predicted medication adherence and which subscales significantly predicted adherence. To do this, each culturally adapted IPQ-R subscale was included as an independent variable. The control variables included patient demographics and clinical variables. The dependent variable was self-reported medication adherence.

#### Internal Consistency (Reliability)

The internal consistency of the culturally adapted IPQ-R was measured using Cronbach alpha values within and across subscales (domains) [37]. Specifically, we examined the Cronbach alpha of the subscales and a stratified alpha across subscales. Cronbach alpha values ≥0.7 were considered to reflect good internal consistency. We evaluated whether the adapted questionnaire showed more multidimensionality with greater internal consistency than the current IPQ-R. Item- to-total correlations were obtained as a basis for item analysis.

#### Test-retest Reliability

Test-retest reliability is a measure of reliability of a questionnaire that is obtained by administering the same questionnaire twice over a period of time to a group of individuals. The stability of the illness beliefs, as evaluated by the culturally adapted questionnaire items, was assessed through test-retest correlations at the subscale level. Higher correlations between the illness perception scores at the initial and 8-week time would suggest more reliability/stability. Pearson correlations were used for this evaluation.

## Results

The baseline survey resulted in 170 respondents with only 29 completing the follow-up survey due to the COVID pandemic occurring during that time. Demographic and clinical characteristics of the participants are reported in Table 1. The participants had an average age of approximately 56 years old and took nearly 2 oral medications. Most responders were female, had a high school education or more, and reported fair health.

**Table 1:** Demographic and clinical characteristics

Variable (***n*** = 170)	Frequency Number (Percentage)	Mean (Standard Deviation)
Age (years)		55.7 (7.2)
Gender (Female)	100 (58.8%)	
Highest Level of Education		
8th grade or less	9 (5.3%)	
Some high school	27 (15.9%)	
High school graduate or GED	47 (27.6%)	
Trade School	3 (1.8%)	
Some College	44 (35.2%)	
Associate’s/Bachelor’s Degree (College Graduate)	22 (15.9%)	
Graduate Degree	14 (8.3%)	
Missing	4 (2.4%)	
Relationship Status		
Married / Legally recognized domestic partnership	36 (21.2%)	
Living with a partner	10 (5.9%)	
Divorced or separated	33 (19.4%)	
Widowed	10 (5.9%)	
Single, never married	78 (45.9%)	
Missing	3 (1.8%)	
Number of oral medications		1.87 (1.2)
Perceived Health Status		
Excellent	6 (3.5%)	
Very good	16 (9.4%)	
Good	59 (34.7%)	
Fair	71 (41.8%)	
Poor	13 (7.6%)	
Missing	5 (2.9%)	

### Content Validity

After expert review, 29 of the 44 items were tested using the cognitive interviewing process. Five problematic items were identified and corrected. Details of this process have been published previously [19].

### Construct Validity

Items that were oppositely worded (i.e., items that needed reverse scoring) had poor inter-correlations and were removed from the analysis due to the apparent confusion created for many respondents. As the response scale for the causes and identity domains are distinct from the 5-point Likert scale used for all other domains, the exploratory factor analysis did not include items from the causes and identity domains. Hence, only 7 domains were included. After removing the poorly performing items, a new 9-factor structure for the culturally adapted questionnaire emerged. Unlike the expected 8-factor structure (7 + 1 new sociocultural domain), the new factor structure of the culturally adapted IPQ-R was distinct from the old factor structure of the IPQ-R. The ‘consequences’ domain from the IPQ-R emerged as two factors (external and internal consequences) in the new factor structure of the culturally adapted IPQ-R while the ‘emotional representations’ domain in the IPQ-R emerged as separate ‘present’ and ‘future’ emotional representation factors in the new factors structure of the adapted IPQ-R. ‘Personal control’, ‘treatment control’ and one item in the ‘sociocultural influences’ domain together formed the ‘control’ domain in the new structure of the adapted IPQ-R. ‘Illness coherence’ was differently conceptualized as ‘illness interpretations’ to capture more accurately the additional culturally-adapted items within this domain in the new factor structure. This new description represented an active process of interpreting the illness, rather than just understanding it. Timeline and ‘Timeline cyclical’ domains remained unchanged with no new items added to it. Most items had factor loadings greater than 0.4 except 10 items that were lesser. We only had three items that were double loaded with low loadings (< 0.3) on both factors. The old and new factor structure with the factor loadings of all items is included in Table 2. The full factor-loading matrix is included in the Appendix as a supplementary file. The factor score correlations (Table 3) were not high (less than or equal to 0.5), indicating that the 9 factors were distinct from each other. In choosing the final factor solution, we balanced consideration of the eigenvalues, as suggested by a scree plot, as well as theoretical interpretability of the factors.

**Table 2:** Old and new factor structure with factor loadings

Old Domains [7]	New Domains [9]	Old and Adapted/New Items^a^	Factor Loading
Consequences	**External Consequences** [(EXT) CON]: (relationships/work/family)	1. My diabetes reduces the control I have over my life.	.287
		2. My diabetes has harmed my relationship with others close to me.	.820
		3. My diabetes has caused difficulties in my relationships with family and friends.	.941
		4. My diabetes has caused my relationships with family and friends to be less close.	.866
		5. My diabetes reduces my participation in social activities within the community.	.384
	**Internal Consequences** [(PL)CON]: personal life consequences	6. My diabetes takes away the ability to enjoy food in my daily life.	.347
		7. My diabetes has taken away my ability to eat the food I grew up eating.	.478
		8. My diabetes is a serious condition.	.816
		9. My diabetes has major consequences on my life.	.681
Personal Control	**Control** [CONT (PC&TC)]: PC refers to control through family and self, while TC refers to control through medications	**PC**	
		10. My friends and family encourage me to manage my diabetes.	.558
		11. I have the power to influence my diabetes.	.486
Treatment Control		**TC**	
		12. Medications can help with my diabetes.	.472
		13. Medications can help me survive with my diabetes.	.473
		14. My treatment will be effective in curing my diabetes.	.512
		15. The negative effects of my diabetes can be prevented (avoided) by my treatment.	.526
		16. My treatment can control my diabetes.	.328
SCI		**17. SCI:** As a black person, I have to advocate for myself if I want to live with diabetes	.428
Illness Coherence	**Illness Interpretation** (II) Active process of interpretation of illness	18. I understand how I get diabetes.	.248 .405
		19. I have a clear picture or understanding of my condition.	
		20. It is important not to worry about my diabetes so as to protect my physical and mental health.	.315
		21. Faith in God helps control my diabetes.	.441
		22. God helps me not to worry about my diabetes.	.457
Emotional Representations	**Present Emotional Representations** (ERp): current feelings and worries	23. It is hard for me to accept that I have diabetes.	.455
		24. It makes me mad that I have to change my life because of diabetes.	.721
		25. I am frustrated while having diabetes.	.718
		26. I am depressed because I have diabetes.	.726
		27. My diabetes controls my life.	.536
		28. I am upset I have diabetes.	.547
		29. I am concerned about dying from my diabetes.	.357
		30. I am worried about my children/grandchildren getting diabetes.	.562
		31. I get depressed when I think about my diabetes.	.894
		32. When I think about my diabetes I get upset.	1.023
		33. My diabetes makes me feel angry.	.947
		34. Having this diabetes makes me feel anxious.	.526
		35. My diabetes makes me feel afraid.	.608
	**Future Emotional Representation**s (ERf): Worries about future complications & outcomes	36. I am scared of having complications from my diabetes.	.560
		37. The experiences of my family and friends has led me to fear diabetes complications.	.602
		38. Having diabetes makes me worry about my future.	.603
		39 I am worried my diabetes will stop me from seeing my children and grandchildren grow up.	.617
None	**Sociocultural Influences** (SCI):	40. Being Black decreases my chances of knowing about diabetes control.	.830
		41. Being Black reduces my chances of getting information about diabetes.	.982
		42. Being Black makes me more likely to get diabetes.	.436
		43. Diabetes is a disease not discussed within the Black community.	.355
		44. My friends and family discourage me from being open about my diabetes.	.341
		45. Being poor contributed to my getting diabetes.	.479
Timeline (Acute/Chronic)	**Timeline**	46. My diabetes is likely to be permanent rather than temporary.	.577
		47. My diabetes will last for a long time.	.957
		48. I expect to have diabetes for the rest of my life.	.751
		49. Nothing can make my diabetes go away.	.188
Timeline Cyclical	**Timeline Cyclical**	50. The symptoms of my diabetes change a great deal from day to day.	.778
		51. My symptoms come and go in cycles.	.695
		52. My diabetes is very unpredictable.	.691
		53. I go through cycles in which my diabetes gets better and worse.	.412
		54. My diabetes is a big part of who I am.	.332

Extraction Method: Maximum Likelihood. Rotation Method: Promax with Kaiser Normalization

^a^Items with poor inter-correlations not included

**Table 3:** Factor Score Correlations

Factor	1	2	3	4	5	6	7	8	9
1	1.000	.444	.531	.563	−.161	.187	.536	.372	.061
2	.444	1.000	.450	.375	−.311	.327	.182	.307	.130
3	.531	.450	1.000	.367	−.242	.225	.341	.248	.117
4	.563	.375	.367	1.000	−.130	.174	.403	.408	.217
5	−.161	−.311	−.242	−.130	1.000	.032	.009	.075	.000
6	.187	.327	.225	.174	.032	1.000	.175	.352	.050
7	.536	.182	.341	.403	.009	.175	1.000	.248	.055
8	.372	.307	.248	.408	.075	.352	.248	1.000	−.035
9	.061	.130	.117	.217	.000	.050	.055	−.035	1.000

Extraction Method: Maximum Likelihood. Rotation Method: Promax with Kaiser Normalization

### Convergent validity

The Necessity and Concern beliefs domains of the Beliefs in Medicines questionnaire significantly correlated with most of the new domains of the culturally adapted questionnaire. The Pearson’s correlation values were also not greater than 0.7, indicating good convergent validity without too much overlap between the questionnaires. The correlation values of the old and new domains with Beliefs in Medicines domains are presented in Table 4.

**Table 4:** Correlations with beliefs in medicines scale for the old and new domains

Old Domains [7]	Pearson’s Correlation Necessity Beliefs	Pearson’s Correlation Concern Beliefs	New Domains [9]	Pearson’s Correlation Necessity Beliefs	Pearson’s Correlation Concern Beliefs
Consequences	0.274**	0.428**	External Consequences	0.211**	0.393**
			Internal Consequences	0.256**	0.276**
Personal Control	0.159*	0.055	Control	0.221**	−0.007
Treatment Control	0.073	−0.180*			
Illness Coherence	0.014	−0.313**	Illness Interpretation	0.072	0.153*
Emotional Representations	0.337**	0.634**	Present Emotional Representations	0.314**	0.634**
			Future Emotional Representations	0.317**	0.515**
None			Sociocultural Influences	0.142	0.526**
Timeline	0.279**	0.209**	Timeline	0.266**	0.302**
Timeline Cyclical	0.293**	0.429**	Timeline Cyclical	0.326**	0.439**

*means, signficant at *p*<0.05 and **means signficant at *p*<0.01

### Predictive validity

The culturally adapted IPQ-R significantly predicted medication adherence. However, only the timeline and timeline cyclical domains individually predicted medication adherence, after controlling for patient demographic factors and clinical characteristics. The results of the regression model including the zero order correlations between the subscales and adherence are presented in Table 5.

**Table 5:** Regression model of the culturally-adapted IPQ-R predicting medication adherence (*n* = 165)

Independent Variables	Standardized Coefficient (β)	t-test value	Pearson’s Correlations with Adherence
R^2^ = 0.33, *p* < 0.001			
Age	0.20	2.87**	0.274**
Gender	0.04	0.67	0.05
Overall Health	−0.05	−0.75	0.07
External Consequences	0.12	1.40	0.37**
Internal Consequences	−0.14	−1.63	0.13*
Control	−0.05	−0.72	− 0.11
Illness Interpretation	−0.05	− 0.73	−0.02
Present Emotional Representations	0.18	1.69	0.40**
Future Emotional Representations	−0.05	− 0.48	0.25**
Sociocultural Influences	0.16	1.95	0.38**
Timeline	0.17	2.20*	0.34**
Timeline Cyclical	0.18	2.04*	0.34**

*p < 0.05 **p < 0.01

### Internal consistency

All domains except the illness interpretation domain of the culturally adapted IPQ-R had Cronbach’s alpha values higher than 0.7, indicating good internal consistency. The internal consistency of the new domains was better than the internal consistency of the old domains. The internal consistency results are presented in Table 6. Most item-total correlations (Table 7) were significant, indicating good reliability of items.

**Table 6:** Internal consistency of domains according to the old and new factor structure

Old Domains [7]	Cronbach’s Alpha	New Domains [9]	Cronbach’s Alpha
Consequences	0.851	External Consequences	0.832
		Internal Consequences	0.730
Personal Control	0.323	Control	0.701
Treatment Control	0.606		
Illness Coherence	0.697	Illness Interpretation	0.561
Emotional Representations	0.924	Present Emotional Representations	0.930
		Future Emotional Representations	0.830
None	N/A	Sociocultural Influences	0.778
Timeline (Acute/Chronic)	0.646	Timeline	0.724
Timeline Cyclical	0.765	Timeline Cyclical	0.758

**Table 7:** Item-total bivariate correlations of domains according to the old and new factor structure

Old Domains and Items – Pearson Correlations	New Domains and Items - Pearson Correlations
**Consequences**	**External Consequences**
1. 0.606**	1. 0.618**
2. 0.718**	2. 0.836**
3.0.753**	3. 0.874**
4. 0.713**	4. 0.840**
5..0.660**	5. 0.696**
	**Internal Consequences**
6. 0.659**	6. 0.743**
7. 0.710**	7. 0.773**
8. 0.615**	8. 0.747**
9. 0.388**	9. 0.707**
10. 0.470**	
11. 0.676**	
**Personal Control**	**Control**
1. 0.431**	10. 0.536**
2. 0.017	11. 0.513**
3. 0.342**	12. 0.483**
4. 0.574**	13. 0.312
5. 0.543**	14. 0.438**
6. 0.501**	15. 0.331
7. 0.373**	16. 0.107
8. 0.370**	17. 0.333
9. 0.383**	
**Treatment Control**	
1. 0.541**	
2. 0.551**	
3. 0.472**	
4. 0.459**	
5. 0.602**	
6. 0.609**	
7. 0.575**	
**Illness Coherence**	**Illness Interpretation**
1. 0.713**	18. 0.495**
2. 0.580**	19. 0.529**
3. 0.795**	20. 0.654**
4. 0.742**	21. 0.689**
5. 0.520**	22. 0.636**
**Emotional Representations**	**Present Emotional Representations**
1. 0.661**	23. 0.671**
2. 0.621**	24. 0.763**
3. 0.682**	25. 0.746**
4. 0.644**	26. 0.816**
5. 0.700**	27. 0.711**
6. 0.740**	28. 0.676**
7. 0.726**	29. 0.683**
8. 0.800**	30. 0.574**
9. 0.693**	31. 0.845**
10 0.635**	32. 0.865**
11. 0.153*	33. 0.819**
12. 0.692**	34. 0.608**
13. 0.563**	35. 0.803**
14. 0.824**	
15. 0.815**	**Future Emotional Representations**
16. 0.764**	36. 0.807**
17. 0.249**	37. 0.823**
18. 0.576**	38. 0.835**
19. 0.789**	39. 0.789**
**None**	**Sociocultural Influences**
	40. 0.808**
	41. 0.792**
	42. 0.622**
	43. 0.620**
	44. 0.550**
	45. 0.739**
**Timeline (Acute/Chronic)**	**Timeline**
1. 0.625**	46. 0.730**
2. 0.450**	47. 0.841**
3. 0.305**	48. 0.838**
4. 0.569**	49. 0.549**
5. 0.341**	
6. 0.580**	
7. 0.739**	
8. 0.677**	
**Timeline Cyclical**	**Timeline Cyclical**
1. 0.789**	50. 0.766**
2. 0.751**	51. 0.715**
3. 0.802**	52. 0.785**
4. 0.719**	53. 0.680**
	54. 0.620**

** Significant at *p* < 0.01

### Test-retest reliability

Baseline and follow-up Pearson’s correlation values were significant for all domains except ‘illness interpretation’ and ‘sociocultural influences.’ None of the correlation values were higher than 0.7, indicating moderate reliability. However, the results should be interpreted cautiously considering the small sample size of 29 respondents for the follow-up survey, limited by data collection restrictions of the COVID-19 pandemic. The results are presented in Table 8.

**Table 8:** Baseline and follow-up correlation according to new factor structure (*n* = 29)

Domain	Baseline & Follow-up Pearson’s Correlation
External Consequences	0.392*
Internal Consequences	0.607*
Control	0.606*
Illness Interpretation	−0.064
Present Emotional Representations	0.473**
Future Emotional Representations	0.459*
Sociocultural Influences	0.174
Timeline	0.635**
Timeline Cyclical	0.459*

** *p* < 0.01 and * *p* < 0.05

## Discussion

To our knowledge, this is the first study to examine the cultural adaptation of the Illness Perception Questionnaire-Revised for use in a US sample of African Americans and investigate the reliability and validity of this adapted questionnaire. The results of the study showed that the culturally adapted IPQ-R for African Americans with diabetes may have preliminary construct, convergent and predictive validity, and reliability.

The new factor structure of the culturally adapted IPQ-R was distinct from the original IPQ-R. The original ‘consequences’ domain was represented as two factors (external and internal consequences) in the new structure while ‘emotional representations’ domain was represented as ‘present’ and ‘future’ factors. ‘Personal control’, ‘treatment control’ and one item originally part of the ‘sociocultural influences’ domain together formed the ‘control’ domain. ‘Illness coherence’ was conceptualized as ‘illness interpretations’ to more accurately capture the additional culturally-adapted items within this domain that represented an active process of interpreting the illness than a static understanding. As well, we observed a new sociocultural domain that further delineates the unique perceptions of diabetes representations among African Americans. This new factor structure reflects the limitations of the IPQ-R among African Americans, reported by authors who showed low reliability of the same IPQ-R subscales (timeline, consequences, control, illness coherence, and emotional representation), and stated that “findings of beliefs in these areas should be interpreted cautiously” [17]. In addition, they questioned the cultural appropriateness of the IPQ-R with African Americans, due to the psychometric problems of the questionnaire when used with African Americans.

We discuss some of the similarities and differences between the original IPQ-R and the adapted version. Regarding the construct validity of the adapted measure, it was interesting to see that the factor structure we expected was not observed. We projected that since the original factor structure of the IPQ-R was a 7-factor structure, by adding the new sociocultural domain, we would end up with an 8-factor structure. Instead, we observed a new 9-factor structure for the culturally adapted measure that separated the IPQ-R domain, ‘consequences’, into external and internal consequences, as well as ‘emotional representations’ into present and future emotional representations. There are some reasons why this may have occurred as discussed below.

It is possible that for African Americans with diabetes, there is more depth to the way diabetes is perceived and how it affects their social and environmental contexts, including relationships, family, work, etc., compared to other racial/ethnic groups. For example, our initial exploratory qualitative work showed how diabetes may have affected the relationship with family and friends, including the stigmatizing effect of the disease in the community [7, 8]. This factor may not be characteristic of Western European populations upon which the original IPQ-R was validated. Prior studies show that African Americans have strong social norms and community influences that impact their health practices, self-care and possibly illness perceptions [38]. As well, there is evidence for the role of family support in African Americans self-management of chronic illnesses like diabetes [38, 39]. Our initial qualitative data from the parent study also indicated that illness perceptions may play a role in medication adherence [40]. Therefore, a culturally adapted questionnaire that captures the sociocultural influence of family and community is reflected in the new factor-structure. This might explain the differences in factor structure between the original IPQ-R and the adapted version as the former scale might have not captured the in-depth cultural influences affecting the perceptions of diabetes, observed qualitatively among African Americans.

Related to the ‘consequences’ domain, the internal consequences domain in the adapted IPQ-R reflects how food is represented within African Americans culture. Prior studies have shown how dietary options based on cultural background are important modifications to make in nutrition and lifestyle education [41–43]. Since diabetes self-management usually includes making dietary changes, it is not surprising that African Americans’ perceptions of diabetes reflect the notion of how the illness affects their ability to enjoy culturally relevant meal options [44]. Aside from food, the factor structure of the culturally adapted IPQ-R may reflect the burden of diabetes on African Americans compared to other racial/ethnic groups, which may not have been captured in the IPQ-R [41, 42].

In the new factor structure, we observe how the perception of diabetes control among African Americans is captured in other ways besides self-control, i.e., control is influenced by the family and the need to increase self-agency to stay in control of diabetes. This need for self-agency reflects the prior and current marginalization, lower social position, and lack of self-agency that African Americans experience [45, 46]. These factors are possibly captured in the need for self-advocacy to improve disease self-management.

Instead of emotional representations due to the IPQ-R captured in one domain, the new factor structure of the culturally adapted IPQ-R captures the underlying layers of emotional responses to having diabetes among African Americans in current instances and in the future. Though African Americans with diabetes currently experience the burden of diabetes in their life, they also think beyond their current situation to future worries, such as intergenerational effect regarding how the disease may affect their children and grandchildren. The tight knit community in most African American households and the implications of diabetes in the family are reflected in their perceptions of diabetes and are characterized in the new factor structure of the culturally adapted IPQ-R [47, 48].

It was interesting to observe how illness coherence is conceptualized differently among African Americans with diabetes in the new factor structure. The original IPQ-R captures a patient’s understanding of an illness. However, the culturally adapted IPQ-R captured a more active process of possible interpretation of the disease, beyond a static understanding of it. Hence, we see the unique sociocultural influences of religion and faith represented in African Americans worry and control of diabetes, which is captured in the illness interpretation factor [9]. This might explain the differences in the factor structure of both the original and adapted IPQ-R. While the original IPQ-R is able to capture a broad understanding of the perception of an illness, the adapted IPQ-R further explores the cultural nuances within the understanding of an illness.

Though unique sociocultural influences on African Americans’ perceptions of diabetes are reflected throughout the culturally adapted IPQ-R, we also observe a specific domain that captures the racial identity of what it means to be African American/Black, the underlying impact of racial discrimination and how this influences diabetes knowledge, perception of disease susceptibility, stigma of diabetes within the community, and the influence of socioeconomic status in the development of diabetes. Prior studies have reported the perception of racism influencing the perception of illnesses such as diabetes and hypertension [48, 49]. We perceive that the newly included sociocultural domain in the culturally adapted IPQ-R recognizes the influence of these important perceptions, based on African Americans’ lived experiences, which was not represented in the original IPQ-R.

Our findings showed good preliminary convergent and predictive validity of the culturally adapted IPQ-R based on the association of illness perceptions with beliefs in medicines and medication adherence. Prior studies evaluated the predictive validity of the original IPQ-R, by evaluating the relationship of the illness constructs with medication adherence (behavioral outcome) or myocardial infarction (co-morbidity) in patients with diabetes [14], fatigue severity and sickness impact in patients with multiple sclerosis [13], attitudes and intentions towards preventive behaviors in healthy individuals [50], and depression in cancer patients [51]. This exploratory psychometric investigation shows that the adapted IPQ-R is better applicable to African Americans’ culturally influenced beliefs about diabetes and should be considered when their illness perceptions are assessed in behavioral interventions that target self-management behaviors like medication adherence.

Many African Americans do not strongly accept the biomedical explanations for chronic diseases like diabetes but often attribute factors outside of their personal control in disease causality [8, 52, 53]. AUTHOR et al., 2009 further showed how the personal control sub-scale of the IPQ-R when used with African Americans with mental illness showed low reliability, indicating a lack of validity of its use with African Americans [16]. The culturally adapted IPQ-R may assess these unique beliefs about diabetes in African Americans, which can have a significant impact on self-management behaviors, like medication adherence, which are necessary for diabetes control and quality of life.

The strengths of this study are the use of a rigorous exploratory, mixed methods design to culturally adapt the IPQ-R for African Americans with diabetes. In addition, to our knowledge, this is the first study to explore and consider how to improve the cultural appropriateness of the IPQ-R among African American/Blacks with diabetes. We report several study limitations, primarily, our sample may not be representative of all Black/African American ethnicities, as we did not collect specific information on the ethnic backgrounds of our sample population. Due to the COVID-19 pandemic, face-to-face research was stopped, and alternate modes of surveys did not yield responses. The test-retest reliability results should be interpreted with caution until similar tests can be conducted with larger samples. Also, in this questionnaire only initial perceptions of diabetes were captured among a middle age group of African Americans living in a Midwestern city in the US, which may not reflect the perceptions of diabetes among the general population of African Americans with diabetes. Future iterations of the study must be conducted with African American populations from different regions, ethnicities, and age groups. To reduce responder burden, we minimized the length of the survey by not including every IPQ-R item. We included the unchanged original IPQ-R items but excluded items that were changed/reworded during the development process of the culturally adapted IPQ-R. This meant that we could not individually compare each original item with its adapted version. A future study will consider the inclusion of all items in both the IPQ-R and culturally adapted IPQ-R with a larger sample of African American populations, including a wider representative group. The testing of the questionnaire using confirmatory analytical approaches will be considered in a future study.

## Conclusion

This study provides preliminary support for the validity of the culturally adapted IPQ-R in African Americans with diabetes, showing good construct, convergent and predictive validity, as well as reliability. With further rigorous testing using confirmatory factor analytical approaches and a representative sample of African Americans including immigrant Blacks from different ethnic backgrounds, this culturally adapted IPQ-R may be used to accurately assess diabetes illness perceptions in African Americans. The addition of a new subscale in the culturally adapted IPQ-R, highlighting the sociocultural influence of African Americans’ illness perceptions, may be based on African Americans’ lived experiences. Upon further testing of the adapted IPQ-R, the survey might be better in identifying specific illness perceptions that influence patient health behaviors. Accurate characterizations of illness perceptions in African Americans are important in further understanding the mechanistic pathways of illness perceptions in self-management behaviors like medication adherence and using the construct as an intervention target for psychosocial and behavioral interventions.

## Supplementary Information

**Additional file 1.** Full 9 factor structure. The full factor loading matrix of the old and new survey items.

## Data Availability

The dataset used and/or analyzed for the current study are available from the corresponding author on reasonable request.

## Abbreviations

IPQ-R: Illness Perception Questionnaire- Revised
COVID-19: Coronavirus Disease 2019

## References

[1] Control CfD, Prevention (2011). National diabetes fact sheet: national estimates and general information on diabetes and prediabetes in the United States, 2011.

[2] Marshall MC (2005). diabetes in African Americans. Postgrad Med J.

[3] Bhattacharya G (2012). Self-management of type 2 diabetes among African Americans in the Arkansas Delta: a strengths perspective in social-cultural context. J Health Care Poor Underserved.

[4] Hu D, Juarez DT, Yeboah M, Castillo TP (2014). Interventions to increase medication adherence in African-American and Latino populations: a literature review. Hawai'i J Med Public Health.

[5] Skelly AH, Dougherty M, Gesler WM, Soward AC, Burns D, Arcury TA (2006). African American beliefs about diabetes. West J Nurs Res.

[6] Patel I, Erickson SR, Caldwell CH, Woolford SJ, Bagozzi RP, Chang J (2016). Predictors of medication adherence and persistence in Medicaid enrollees with developmental disabilities and type 2 diabetes. Res Soc Adm Pharm.

[7] Shiyanbola OO, Ward E, Brown C (2018). Sociocultural influences on African Americans’ representations of type 2 diabetes: a qualitative study. Ethnicity Dis.

[8] Shiyanbola OO, Ward EC, Brown CM. Utilizing the common sense model to explore African Americans’ perception of type 2 diabetes: a qualitative study. PloS one. 2018 Nov 21;13(11):e0207692. 10.1371/journal.pone.0207692 PMC6248983 30462704

[9] Ward EC, Mengesha M, Issa F (2014). Older A frican A merican women's lived experiences with depression and coping behaviours. J Psychiatr Ment Health Nurs.

[10] Leventhal H, Nerenz DR, Steele DJ. Illness representations and coping with health threats. In: Handbook of Psychology and Health (Volume IV). Routledge; 2020. p. 219–52.

[11] Kugbey N, Oppong Asante K, Adulai K (2017). Illness perception, diabetes knowledge and self-care practices among type-2 diabetes patients: a cross-sectional study. BMC Res notes.

[12] Weinman J, Petrie KJ, Moss-Morris R, Horne R (1996). The illness perception questionnaire: a new method for assessing the cognitive representation of illness. Psychol Health.

[13] Moss-Morris R, Weinman J, Petrie K, Horne R, Cameron L, Buick D (2002). The revised illness perception questionnaire (IPQ-R). Psychol Health.

[14] Broadbent E, Petrie KJ, Main J, Weinman J (2006). The brief illness perception questionnaire. J Psychosom Res.

[15] Abubakari A-R, Jones MC, Lauder W, Kirk A, Devendra D, Anderson J (2012). Psychometric properties of the Revised Illness Perception Questionnaire: Factor structure and reliability among African-origin populations with type 2 diabetes. Int J Nurs Stud.

[16] Ward EC, Heidrich SM (2009). African American women's beliefs about mental illness, stigma, and preferred coping behaviors. Res Nurs Health.

[17] Ward E, Wiltshire JC, Detry MA, Brown RL (2013). African American men and women's attitude toward mental illness, perceptions of stigma, and preferred coping behaviors. Nurs Res.

[18] Calvin D, Quinn L, Dancy B, Park C, Fleming SG, Smith E (2011). African Americans’ perception of risk for diabetes complications. Diabetes Educ.

[19] Shiyanbola OO, Bolt D, Tarfa A, Brown C, Ward E (2019). A content validity and cognitive interview process to evaluate an Illness Perception Questionnaire for African Americans with type 2 diabetes. BMC Res Notes..

[20] Shiyanbola OO, Rao D, Bolt D, Brown C, Zhang M, Ward E (2021). Using an exploratory sequential mixed methods design to adapt an Illness Perception Questionnaire for African Americans with diabetes: the mixed data integration process. Health Psychol Behav Med.

[21] Horne R, Weinman J, Hankins M (1999). The beliefs about medicines questionnaire: the development and evaluation of a new method for assessing the cognitive representation of medication. Psychol Health.

[22] Kripalani S, Risser J, Gatti ME, Jacobson TA (2009). Development and evaluation of the Adherence to Refills and Medications Scale (ARMS) among low-literacy patients with chronic disease. Value Health.

[23] Stapleton CD (1997). Basic Concepts in Exploratory Factor Analysis (EFA) as a Tool To Evaluate Score Validity: A Right-Brained Approach.

[24] De Winter JC, Dodou D (2012). Factor recovery by principal axis factoring and maximum likelihood factor analysis as a function of factor pattern and sample size. J Appl Stat.

[25] Jiang H, Zhao L, Yang L, Cai H (2017). Relationships among illness perceptions, medication beliefs and medication adherence in primary angle closure glaucoma patients. Zhonghua yan ke za Zhi.

[26] Ross S, Walker A, MacLeod MJ (2004). Patient compliance in hypertension: role of illness perceptions and treatment beliefs. J Hum Hypertens.

[27] Chin C-L, Yao G, Michalos AC (2014). Convergent Validity. Encyclopedia of Quality of Life and Well-Being Research.

[28] Schuler H, Smelser NJ, Baltes PB (2001). Personnel Selection, Psychology of. International Encyclopedia of the Social & Behavioral Sciences.

[29] Wainer H, Sireci SG, Kempf-Leonard K (2005). Item and Test Bias. Encyclopedia of Social Measurement.

[30] Aflakseir A (2012). Role of illness and medication perceptions on adherence to medication in a group of Iranian patients with type 2 diabetes. J Diabetes.

[31] Searle A, Norman P, Thompson R, Vedhara K (2007). A prospective examination of illness beliefs and coping in patients with type 2 diabetes. Br J Health Psychol.

[32] Baroletti S, Dell'Orfano H (2010). Medication adherence in cardiovascular disease. Circulation..

[33] Iskandarsyah A, de Klerk C, Suardi DR, Sadarjoen SS, Passchier J (2014). Consulting a traditional healer and negative illness perceptions are associated with non-adherence to treatment in Indonesian women with breast cancer. Psycho Oncol.

[34] Olszanecka-Glinianowicz M, Almgren-Rachtan A (2014). The adherence and illness perception of patients diagnosed with asthma or chronic obstructive pulmonary disease treated with polytherapy using new generation Cyclohaler. Postepy Dermatologii i alergologii.

[35] Saarti S, Hajj A, Karam L, Jabbour H, Sarkis A, El Osta N (2016). Association between adherence, treatment satisfaction and illness perception in hypertensive patients. J Hum Hypertens.

[36] Thomson P, Rushworth GF, Andreis F, Angus NJ, Mohan AR, Leslie SJ (2020). Longitudinal study of the relationship between patients’ medication adherence and quality of life outcomes and illness perceptions and beliefs about cardiac rehabilitation. BMC Cardiovasc Disord.

[37] Taber KS (2018). The Use of Cronbach’s Alpha When Developing and Reporting Research Instruments in Science Education. Res Sci Educ.

[38] Miller TA, DiMatteo MR (2013). Importance of family/social support and impact on adherence to diabetic therapy. Diabetes Metab Syndrome Obes.

[39] Routh B, Hurt T, Winham D, Lanningham-Foster L (2019). Family legacy of diabetes-related behaviors: An exploration of the experiences of African American parents and adult children. Global Qual Nurs Res.

[40] Shiyanbola OO, Brown CM, Ward EC (2018). “i did not want to take that medicine”: African-Americans’ reasons for diabetes medication nonadherence and perceived solutions for enhancing adherence. Patient Preference Adherence.

[41] Batts ML, Gary TL, Huss K, Hill MN, Bone L, Brancati FL (2001). Patient priorities and needs for diabetes care among urban African American adults. Diabetes Educ.

[42] Chesla CA, Fisher L, Mullan JT, Skaff MM, Gardiner P, Chun K (2004). Family and disease management in African-American patients with type 2 diabetes. Diabetes Care.

[43] Liburd LC (2003). Food, identity, and African-American women with type 2 diabetes: an anthropological perspective. Diabetes Spectrum.

[44] Rao D, Meyer J, Maurer M, Shiyanbola OO (2021). Perceptions of psychosocial and interpersonal factors affecting self-management behaviors among African Americans with diabetes. Explor Res Clin Soc Pharm.

[45] Baah FO, Teitelman AM, Riegel B (2019). Marginalization: Conceptualizing patient vulnerabilities in the framework of social determinants of health—An integrative review. Nurs Inq.

[46] Mays VM, Cochran SD, Barnes NW (2007). Race, race-based discrimination, and health outcomes among African Americans. Annu Rev Psychol.

[47] Scollan-Koliopoulos M, O’Connell KA, Walker EA (2005). The first diabetes educator is the family: using illness representation to recognize a multigenerational legacy of diabetes. Clin Nurse Spec.

[48] Wagner JA, Osborn CY, Mendenhall EA, Budris LM, Belay S, Tennen HA (2011). Beliefs about racism and health among African American women with diabetes: a qualitative study. J Natl Med Assoc.

[49] Webb MS, Gonzalez LO (2006). The burden of hypertension: mental representations of African American women. Issues Mental Health NursS.

[50] Figueiras MJ, Alves NC (2007). Lay perceptions of serious illnesses: An adapted version of the Revised Illness Perception Questionnaire (IPQ-R) for healthy people. Psychol Health.

[51] Giannousi Z, Manaras I, Georgoulias V, Samonis G (2010). Illness perceptions in Greek patients with cancer: a validation of the Revised-Illness Perception Questionnaire. Psycho Oncol.

[52] Lukoschek P (2003). African Americans' beliefs and attitudes regarding hypertension and its treatment: a qualitative study. J Health Care Poor Underserved.

[53] Pickett S, Allen W, Franklin M, Peters RM (2014). Illness beliefs in African Americans with hypertension. West J Nurs Res.

