# Supplementary material for: Psychometric evaluation of a culturally adapted illness perception questionnaire for African Americans with type 2 diabetes
Source: BMC Public Health. 2022 Apr 13;22:741. doi: 10.1186/s12889-022-13172-2 (PMC9007270; doi:10.1186/s12889-022-13172-2)
Supplement: Supplementary file 1 — Additional file 1. Full 9 factor structure. The full factor loading matrix of the old and new survey items. [file 12889_2022_13172_MOESM1_ESM.docx]

**Appendix: Full 9 factor structure**

| **Pattern Matrix^a^** | | | | | | | | | |
| --- | --- | --- | --- | --- | --- | --- | --- | --- | --- |
|  | Factor | | | | | | | | |
|  | 1 | 2 | 3 | 4 | 5 | 6 | 7 | 8 | 9 |
| 2b_Nothing can make my diabetes go away._Timeline_New | .025 | -.033 | .296 | .144 | -.274 | .188 | -.047 | -.028 | .021 |
| 2f_My diabetes is likely to be permanent rather than temporary._Timeline_Old | .064 | .139 | .020 | .056 | .045 | .577 | -.072 | -.039 | -.084 |
| 2g_My diabetes will last for a long time._Timeline_Old | .004 | -.009 | -.060 | -.072 | -.077 | .957 | .042 | -.006 | .041 |
| 2h_I expect to have diabetes for the rest of my life._Timeline_Old | -.039 | .086 | .072 | .045 | -.044 | .751 | -.017 | -.054 | -.057 |
| 2i_My diabetes reduces the control I have over my life._Consequences_New | .122 | .287 | -.035 | -.057 | -.154 | .122 | .138 | .193 | .027 |
| 2j_My diabetes has harmed my relationship with others close to me._Consequences_New | -.035 | .820 | .030 | -.014 | .011 | .058 | -.002 | -.079 | .025 |
| 2k_My diabetes has caused difficulties in my relationships with family and friends._Consequences_New | -.050 | .941 | -.013 | -.038 | .071 | .055 | .080 | -.038 | .006 |
| 2l_My diabetes has caused my relationships with family and friends to be less close._Consequences_New | -.045 | .866 | .062 | .077 | .074 | .013 | -.081 | -.051 | -.071 |
| 2m_My diabetes reduces my participation in social activities within the community._Consequences_New | .186 | .384 | -.121 | .174 | -.174 | .046 | -.098 | .026 | .153 |
| 2n_My diabetes takes away the ability to enjoy food in my daily life._Consequences_New | .197 | .066 | .061 | .239 | -.122 | -.097 | -.066 | .347 | .174 |
| 2o_Having diabetes has kept me away from the type of job I want to have._Consequences_New | .142 | .606 | -.067 | .007 | -.222 | -.046 | .088 | -.046 | .064 |
| 2p_My diabetes has taken away my ability to eat the food I grew up eating._Consequences_New | .050 | .071 | .055 | .032 | -.118 | -.135 | .104 | .478 | .169 |
| 2q_My diabetes is a serious condition._Consequences_Old | .019 | -.170 | -.055 | -.113 | -.001 | -.013 | .111 | .816 | .233 |
| 2r_My diabetes has major consequences on my life._Consequences_Old | -.107 | .035 | -.040 | -.007 | .106 | .007 | .179 | .681 | .121 |
| 2s_My diabetes strongly affects the way others see me._Consequences_Old | .097 | .490 | .053 | .126 | .158 | .106 | .041 | .012 | .018 |
| 2t_My diabetes is a big part of who I am._PersonalControl_New | -.053 | -.022 | .084 | .332 | -.027 | .193 | .036 | .171 | **.205** |
| 2v_It is important not to worry about my diabetes so as to protect my physical and mental health._PersonalControl_New | -.019 | .190 | .163 | .113 | .064 | -.117 | -.102 | .187 | .315 |
| 2w_Faith in God helps control my diabetes._PersonalControl_New | -.012 | -.006 | .069 | -.046 | .167 | -.111 | .079 | .227 | .441 |
| 2x_God helps me not to worry about my diabetes._PersonalControl_New | -.142 | .124 | .061 | -.121 | .099 | .050 | .140 | .116 | .457 |
| 3a_My friends and family encourage me to manage my diabetes._PersonalControl_New | .106 | -.118 | .013 | .207 | .558 | .097 | -.038 | -.053 | .179 |
| 3b_I have the power to influence my diabetes._PersonalControl_Old | .018 | .076 | -.036 | .094 | .486 | -.137 | -.163 | .020 | .136 |
| 3e_Medications can help with my diabetes._TreatmentControl_New | -.169 | .109 | .036 | -.080 | .472 | .113 | -.072 | .163 | .248 |
| 3f_Medications can help me survive with my diabetes._TreatmentControl_New | -.074 | .099 | -.077 | .001 | .473 | .035 | .020 | .217 | .072 |
| 3h_My treatment will be effective in curing my diabetes._TreatmentControl_Old | .121 | .066 | .102 | .065 | .512 | -.227 | .075 | -.202 | .031 |
| 3i_The negative effects of my diabetes can be prevented (avoided) by my treatment._TreatmentControl_Old | -.067 | -.102 | .062 | .103 | .526 | -.111 | .155 | -.024 | -.043 |
| 3j_My treatment can control my diabetes._TreatmentControl_Old | -.002 | -.163 | -.162 | .089 | .328 | .125 | .186 | -.094 | .165 |
| 3m_I understand how I get diabetes._IllnessConherence_New | .125 | -.137 | .015 | .084 | .098 | .003 | -.192 | .088 | .248 |
| 3p_I have a clear picture or understanding of my condition._IllnessConherence_Old | .092 | -.042 | -.205 | .089 | .262 | -.007 | -.284 | .119 | .405 |
| 3q_The symptoms of my diabetes change a great deal from day to day._TimelineCyclical_Old | -.024 | .036 | .024 | .778 | .161 | -.100 | .029 | -.039 | -.090 |
| 3r_My symptoms come and go in cycles._TimelineCyclical_Old | -.025 | .032 | -.066 | .695 | .154 | .050 | .018 | -.126 | .027 |
| 3s_My diabetes is very unpredictable._TimelineCyclical_Old | -.079 | .040 | .051 | .691 | -.063 | .015 | .078 | .029 | -.003 |
| 3t_I go through cycles in which my diabetes gets better and worse._TimelineCyclical_Old | .146 | .097 | -.174 | .412 | .116 | .074 | .301 | -.103 | -.109 |
| 3u_I am scared of having complications from my diabetes._EmotionalRepresentations_New | .195 | -.076 | -.001 | .092 | .168 | .053 | .560 | .037 | .031 |
| 3v_The experiences of my family and friends has led me to fear diabetes complications._EmotionalRepresentations_New | .067 | .072 | -.057 | .074 | .096 | -.053 | .602 | .165 | .016 |
| 3w_Having diabetes makes me worry about my future._EmotionalRepresentations_New | .101 | -.067 | -.127 | .274 | -.062 | -.059 | .603 | .151 | -.102 |
| 3x_I am worried my diabetes will stop me from seeing my children and grandchildren grow up._EmotionalRepresentations_New | .160 | .025 | .023 | -.116 | -.064 | .030 | .617 | .135 | .095 |
| 3y_It is hard for me to accept that I have diabetes._EmotionalRepresentations_New | .333 | .119 | .055 | -.075 | -.083 | -.083 | .455 | .004 | -.073 |
| 4a_It makes me mad that I have to change my life because of diabetes._EmotionalRepresentations_New | .721 | -.019 | .147 | -.060 | .071 | .062 | -.025 | .030 | -.243 |
| 4b_I am frustrated while having diabetes._EmotionalRepresentations_New | .718 | -.066 | .028 | .009 | .051 | .049 | -.096 | .263 | -.334 |
| 4c_I am depressed because I have diabetes._EmotionalRepresentations_New | .726 | .151 | -.042 | .008 | .054 | .016 | .065 | .005 | -.151 |
| 4d_My diabetes controls my life._EmotionalRepresentations_New | .536 | .021 | -.008 | .310 | -.059 | -.003 | -.081 | .017 | -.046 |
| 4e_I am upset I have diabetes._EmotionalRepresentations_New | .547 | .001 | .035 | .066 | -.073 | .077 | -.118 | .191 | -.051 |
| 4g_I am concerned about dying from my diabetes._EmotionalRepresentations_New | .357 | -.123 | .094 | .167 | -.012 | .083 | **.304** | .051 | -.149 |
| 4h_I am worried about my children/grandchildren getting diabetes._EmotionalRepresentations_New | .562 | -.271 | .032 | -.055 | .034 | .141 | .166 | -.048 | .207 |
| 4i_I get depressed when I think about my diabetes._EmotionalRepresentations_Old | .894 | .124 | -.125 | -.122 | -.012 | -.076 | .156 | -.099 | -.015 |
| 4j_When I think about my diabetes I get upset._EmotionalRepresentations_Old | 1.023 | -.047 | -.095 | -.078 | -.020 | .019 | -.027 | -.081 | .127 |
| 4k_My diabetes makes me feel angry._EmotionalRepresentations_Old | .947 | .083 | -.040 | -.061 | .020 | -.096 | -.096 | -.078 | .171 |
| 4m_Having this diabetes makes me feel anxious._EmotionalRepresentations_Old | .526 | .046 | .111 | -.043 | .031 | .054 | .099 | -.220 | .236 |
| 4n_My diabetes makes me feel afraid._EmotionalRepresentations_Old | .608 | -.083 | .137 | .068 | -.013 | -.122 | .191 | .027 | .055 |
| 4o_As a black person, I have to advocate for myself if I want to live with diabetes._SocialculturalDomain | .189 | .042 | .242 | -.141 | .428 | .111 | .072 | -.006 | .085 |
| 4p_Being black decreases my chances of knowing about diabetes control._SocialculturalDomain | .082 | .071 | .830 | -.102 | .037 | -.026 | -.016 | -.040 | .149 |
| 4q_Being black reduces my chances of getting information about diabetes._SocialculturalDomain | -.057 | -.023 | .982 | .038 | .090 | .026 | -.152 | -.073 | -.035 |
| 4r_Being black makes me more likely to get diabetes._SocialculturalDomain | .133 | -.024 | .436 | -.145 | .082 | .035 | .050 | .197 | -.073 |
| 4s_Diabetes is a disease not discussed within the black community._SocialculturalDomain | -.089 | -.008 | .355 | .227 | -.019 | .087 | .044 | .115 | .054 |
| 4u_My friends and family discourage me from being open about my diabetes._SocialculturalDomain | -.006 | -.071 | .341 | .304 | -.190 | -.080 | .023 | -.263 | .045 |
| 4w_Being poor contributed to my getting diabetes._SocialculturalDomain | .031 | .057 | .479 | -.012 | -.116 | -.080 | .178 | .004 | .039 |
| Extraction Method: Maximum Likelihood.  Rotation Method: Promax with Kaiser Normalization. | | | | | | | | | |
| 1. Rotation converged in 13 iterations. | | | | | | | | | |
